# 3-(6-Meth­oxy-2-naphth­yl)-1-(2-pyrid­yl)prop-2-en-1-one

**DOI:** 10.1107/S1600536810016880

**Published:** 2010-06-09

**Authors:** Xiang-biao Li

**Affiliations:** a Yibin Vocational & Technical College, Si chuan, People’s Republic of China

## Abstract

There are two mol­ecules in the asymmetric unit of the title compound, C_19_H_15_NO_2_, in which the dihedral angles between the naphthalene ring system and the pyridine ring are 40.5 (3) and 41.2 (4)°. In the crystal, C—H⋯O hydrogen bonds link the mol­ecules.

## Related literature

For medicinal background, see: Petrov *et al.* (2006 ▶).
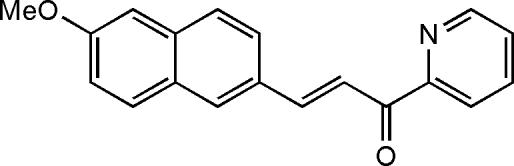


## Experimental

### 

#### Crystal data

C_19_H_15_NO_2_
*M* *_r_* = 289.32
Orthorhombic, 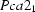

*a* = 7.8560 (16) Å
*b* = 11.542 (2) Å
*c* = 32.353 (7) Å
*V* = 2933.6 (10) Å^3^
*Z* = 8
Mo *K*α radiation
μ = 0.09 mm^−1^
*T* = 293 K
0.20 × 0.10 × 0.10 mm

#### Data collection

Enraf–Nonius CAD-4 diffractometer
5329 measured reflections
5329 independent reflections
2245 reflections with *I* > 2σ(*I*)
*R*_int_ = 0.067
3 standard reflections every 200 reflections  intensity decay: 1%

#### Refinement

*R*[*F* ^2^ > 2σ(*F* ^2^)] = 0.071
*wR*(*F* ^2^) = 0.080
*S* = 1.01
5329 reflections
397 parameters
4 restraints
H-atom parameters constrained
Δρ_max_ = 0.16 e Å^−3^
Δρ_min_ = −0.18 e Å^−3^

### 

Data collection: *CAD-4 Software* (Enraf–Nonius, 1989 ▶); cell refinement: *CAD-4 Software*; data reduction: *XCAD4* (Harms & Wocadlo, 1995 ▶); program(s) used to solve structure: *SHELXS97* (Sheldrick, 2008 ▶); program(s) used to refine structure: *SHELXL97* (Sheldrick, 2008 ▶); molecular graphics: *ORTEP-3* (Farrugia, 1997 ▶); software used to prepare material for publication: *SHELXL97*.

## Supplementary Material

Crystal structure: contains datablocks global, I. DOI: 10.1107/S1600536810016880/hb5431sup1.cif

Structure factors: contains datablocks I. DOI: 10.1107/S1600536810016880/hb5431Isup2.hkl

Additional supplementary materials:  crystallographic information; 3D view; checkCIF report

## Figures and Tables

**Table 1:** Hydrogen-bond geometry (Å, °)

*D*—H⋯*A*	*D*—H	H⋯*A*	*D*⋯*A*	*D*—H⋯*A*
C38—H38*B*⋯O1^i^	0.96	2.53	3.356 (10)	143

Symmetry code: (i) 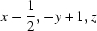.

**Fig. 1.**
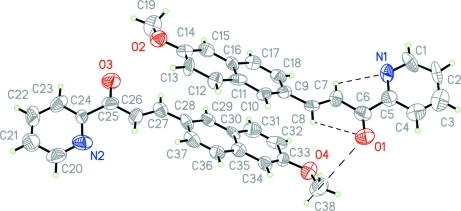
Ellipsoid plot.

## supplementary crystallographic information

### Experimental

In the presence of sodium hydroxide, 6-methoxy-2-naphthaldehyde and 1-(pyridin-2-yl)ethanone in liquid ammonia were stirred at 313 K for 4 h. Sodium hydroxide was filtered off and the filtrate was duluted with dichlormethane and washed by brine. The organic phase was dried over anhydrous sodium sulfate, filtered, and concentrated to give crude product which was recrystallised from from dichlormethane to give yellow blocks of (I).

### Refinement

The absolute structure of the title compound is indeterminate in the present refinement. H atoms were positioned geometrically, with C-H = 0.93, 0.97 and 0.96 Å for aromatic, methylene and methyl H, respectively, and constrained to ride on their parent atoms, with U_iso_(H) = xU_eq_(C), where x = 1.5 for methyl H and x = 1.2 for all other H atoms.

### Crystal data

**Table:**

C_19_H_15_NO_2_	*D*_x_ = 1.310 Mg m^−^^3^
*M**_r_* = 289.32	Melting point: 513 K
Orthorhombic, *P**c**a*2_1_	Mo *K*α radiation, λ = 0.71073 Å
Hall symbol: P 2c -2ac	Cell parameters from 25 reflections
*a* = 7.8560 (16) Å	θ = 9–12°
*b* = 11.542 (2) Å	µ = 0.09 mm^−^^1^
*c* = 32.353 (7) Å	*T* = 293 K
*V* = 2933.6 (10) Å^3^	Needle, yellow
*Z* = 8	0.20 × 0.10 × 0.10 mm
*F*(000) = 1216	

### Data collection

**Table:**

Enraf–Nonius CAD-4 diffractometer	*R*_int_ = 0.067
Radiation source: fine-focus sealed tube	θ_max_ = 25.3°, θ_min_ = 1.3°
graphite	*h* = 0→9
ω/2θ scans	*k* = 0→13
5329 Please give correct value measured reflections	*l* = −38→38
5329 independent reflections	3 standard reflections every 200 reflections
2245 reflections with *I* > 2σ(*I*)	intensity decay: 1%

### Refinement

**Table:**

Refinement on *F*^2^	Primary atom site location: structure-invariant direct methods
Least-squares matrix: full	Secondary atom site location: difference Fourier map
*R*[*F*^2^ > 2σ(*F*^2^)] = 0.071	Hydrogen site location: inferred from neighbouring sites
*wR*(*F*^2^) = 0.080	H-atom parameters constrained
*S* = 1.01	*w* = 1/[σ^2^(*F*_o_^2^) + (0.0012*P*)^2^] where *P* = (*F*_o_^2^ + 2*F*_c_^2^)/3
5329 reflections	(Δ/σ)_max_ < 0.001
397 parameters	Δρ_max_ = 0.16 e Å^−^^3^
4 restraints	Δρ_min_ = −0.18 e Å^−^^3^

### Special details

**Table:**

Geometry. All esds (except the esd in the dihedral angle between two l.s. planes) are estimated using the full covariance matrix. The cell esds are taken into account individually in the estimation of esds in distances, angles and torsion angles; correlations between esds in cell parameters are only used when they are defined by crystal symmetry. An approximate (isotropic) treatment of cell esds is used for estimating esds involving l.s. planes.
Refinement. Refinement of F^2^ against ALL reflections. The weighted R-factor wR and goodness of fit S are based on F^2^, conventional R-factors R are based on F, with F set to zero for negative F^2^. The threshold expression of F^2^ > 2sigma(F^2^) is used only for calculating R-factors(gt) etc. and is not relevant to the choice of reflections for refinement. R-factors based on F^2^ are statistically about twice as large as those based on F, and R- factors based on ALL data will be even larger.

### Fractional atomic coordinates and isotropic or equivalent isotropic displacement parameters (Å^2^)

**Table:**

	*x*	*y*	*z*	*U*_iso_*/*U*_eq_	
O1	0.7960 (7)	0.2745 (4)	0.4482 (2)	0.0861 (18)	
O2	0.3909 (6)	0.1159 (4)	0.14036 (15)	0.0620 (14)	
N1	0.8162 (10)	−0.0296 (6)	0.46063 (19)	0.058 (2)	
C1	0.8679 (12)	−0.1063 (8)	0.4882 (3)	0.075 (3)	
H1B	0.8507	−0.1840	0.4819	0.090*	
C2	0.9437 (11)	−0.0822 (9)	0.5249 (3)	0.073 (3)	
H2A	0.9850	−0.1410	0.5418	0.088*	
C3	0.9574 (13)	0.0347 (9)	0.5365 (3)	0.081 (3)	
H3A	0.9990	0.0564	0.5623	0.097*	
C4	0.9069 (13)	0.1144 (8)	0.5081 (2)	0.062 (3)	
H4A	0.9192	0.1928	0.5141	0.075*	
C5	0.8360 (11)	0.0813 (7)	0.4697 (3)	0.059 (2)	
C6	0.7680 (9)	0.1729 (6)	0.4408 (2)	0.058 (2)	
C7	0.6862 (8)	0.1310 (6)	0.40378 (19)	0.0527 (18)	
H7A	0.6472	0.0550	0.4027	0.063*	
C8	0.6656 (9)	0.2015 (6)	0.37045 (19)	0.0478 (19)	
H8A	0.7015	0.2778	0.3735	0.057*	
C9	0.5936 (10)	0.1705 (6)	0.3306 (2)	0.0489 (19)	
C10	0.6365 (9)	0.2318 (6)	0.29665 (17)	0.0415 (18)	
H10A	0.7065	0.2964	0.2992	0.050*	
C11	0.5771 (8)	0.1993 (5)	0.2577 (2)	0.0454 (17)	
C12	0.6205 (8)	0.2602 (6)	0.22134 (19)	0.049 (2)	
H12A	0.6951	0.3225	0.2232	0.059*	
C13	0.5558 (9)	0.2302 (6)	0.18330 (18)	0.054 (2)	
H13A	0.5861	0.2725	0.1600	0.065*	
C14	0.4439 (9)	0.1356 (6)	0.1796 (2)	0.054 (2)	
C15	0.3968 (8)	0.0729 (6)	0.2144 (2)	0.053 (2)	
H15A	0.3228	0.0103	0.2120	0.063*	
C16	0.4615 (8)	0.1045 (6)	0.2532 (2)	0.0433 (17)	
C17	0.4135 (9)	0.0422 (6)	0.2887 (2)	0.0473 (19)	
H17A	0.3393	−0.0203	0.2864	0.057*	
C18	0.4779 (8)	0.0745 (6)	0.3275 (2)	0.052 (2)	
H18A	0.4458	0.0339	0.3511	0.063*	
C19	0.2639 (13)	0.0281 (7)	0.1341 (3)	0.073 (3)	
H19A	0.2370	0.0230	0.1052	0.110*	
H19B	0.1631	0.0478	0.1494	0.110*	
H19C	0.3067	−0.0452	0.1435	0.110*	
O3	−0.0515 (7)	0.2385 (5)	0.11856 (18)	0.0871 (18)	
O4	0.3447 (6)	0.3763 (4)	0.42812 (15)	0.0726 (15)	
N2	−0.0607 (10)	0.5392 (7)	0.1072 (2)	0.072 (2)	
C20	−0.1230 (14)	0.6188 (8)	0.0806 (3)	0.079 (3)	
H20A	−0.1219	0.6963	0.0884	0.094*	
C21	−0.1890 (14)	0.5899 (9)	0.0420 (3)	0.090 (4)	
H21A	−0.2152	0.6475	0.0230	0.107*	
C22	−0.2144 (14)	0.4768 (9)	0.0325 (3)	0.080 (3)	
H22A	−0.2674	0.4554	0.0080	0.096*	
C23	−0.1595 (11)	0.3954 (9)	0.0602 (3)	0.071 (3)	
H23A	−0.1754	0.3169	0.0549	0.085*	
C24	−0.0819 (10)	0.4303 (7)	0.0953 (2)	0.0412 (19)	
C25	−0.0261 (9)	0.3403 (7)	0.1266 (2)	0.059 (2)	
C26	0.0593 (9)	0.3751 (6)	0.16455 (19)	0.060 (2)	
H26A	0.1091	0.4481	0.1660	0.073*	
C27	0.0680 (9)	0.3057 (6)	0.1972 (2)	0.052 (2)	
H27A	0.0192	0.2326	0.1947	0.062*	
C28	0.1465 (8)	0.3335 (6)	0.2362 (2)	0.0470 (19)	
C29	0.1024 (8)	0.2713 (6)	0.2717 (2)	0.0483 (19)	
H29A	0.0288	0.2086	0.2691	0.058*	
C30	0.1640 (8)	0.2993 (5)	0.3108 (2)	0.0452 (18)	
C31	0.1138 (10)	0.2353 (7)	0.3453 (2)	0.071 (3)	
H31A	0.0394	0.1732	0.3423	0.085*	
C32	0.1747 (9)	0.2641 (6)	0.3839 (2)	0.061 (2)	
H32A	0.1413	0.2220	0.4071	0.074*	
C33	0.2896 (8)	0.3591 (6)	0.3880 (2)	0.0485 (19)	
C34	0.3415 (8)	0.4223 (6)	0.3555 (2)	0.0496 (19)	
H34A	0.4181	0.4829	0.3589	0.059*	
C35	0.2762 (8)	0.3942 (6)	0.3156 (2)	0.0474 (18)	
C36	0.3226 (10)	0.4554 (6)	0.2796 (2)	0.054 (2)	
H36A	0.3981	0.5171	0.2820	0.065*	
C37	0.2623 (8)	0.4281 (6)	0.2421 (2)	0.055 (2)	
H37A	0.2964	0.4715	0.2193	0.066*	
C38	0.4780 (14)	0.4655 (7)	0.4339 (3)	0.094 (3)	
H38A	0.5073	0.4705	0.4626	0.141*	
H38B	0.4360	0.5391	0.4245	0.141*	
H38C	0.5771	0.4447	0.4182	0.141*	

### Atomic displacement parameters (Å^2^)

**Table:**

	*U*^11^	*U*^22^	*U*^33^	*U*^12^	*U*^13^	*U*^23^
O1	0.114 (5)	0.051 (3)	0.093 (4)	−0.004 (3)	−0.023 (4)	−0.014 (3)
O2	0.068 (4)	0.059 (3)	0.059 (4)	−0.006 (3)	0.001 (3)	−0.004 (3)
N1	0.076 (5)	0.064 (4)	0.036 (4)	−0.009 (5)	−0.003 (4)	0.025 (3)
C1	0.067 (6)	0.076 (6)	0.082 (7)	0.019 (5)	0.005 (6)	0.047 (5)
C2	0.045 (5)	0.108 (8)	0.067 (6)	0.001 (6)	−0.021 (5)	0.042 (6)
C3	0.059 (7)	0.122 (8)	0.062 (7)	−0.018 (7)	0.014 (5)	0.004 (7)
C4	0.078 (7)	0.066 (6)	0.044 (5)	−0.012 (5)	0.032 (5)	0.008 (5)
C5	0.058 (6)	0.055 (5)	0.064 (6)	−0.006 (5)	−0.003 (5)	0.002 (4)
C6	0.052 (5)	0.050 (5)	0.073 (6)	−0.001 (4)	0.004 (4)	0.000 (4)
C7	0.044 (5)	0.064 (5)	0.050 (4)	−0.003 (4)	−0.008 (4)	0.001 (4)
C8	0.054 (5)	0.040 (4)	0.050 (5)	0.013 (4)	0.006 (4)	−0.011 (4)
C9	0.064 (5)	0.040 (4)	0.043 (4)	0.006 (4)	0.003 (4)	−0.005 (3)
C10	0.047 (4)	0.040 (4)	0.038 (4)	−0.004 (3)	0.011 (4)	−0.004 (3)
C11	0.036 (4)	0.036 (4)	0.064 (5)	0.001 (3)	0.015 (4)	−0.012 (4)
C12	0.038 (4)	0.057 (5)	0.052 (5)	−0.013 (4)	−0.003 (4)	−0.001 (4)
C13	0.072 (5)	0.065 (5)	0.025 (4)	−0.008 (5)	−0.007 (4)	0.014 (4)
C14	0.058 (5)	0.045 (5)	0.058 (5)	0.008 (4)	0.005 (4)	−0.010 (4)
C15	0.054 (5)	0.041 (5)	0.064 (5)	−0.001 (4)	0.003 (4)	0.003 (4)
C16	0.044 (4)	0.042 (4)	0.044 (4)	0.006 (3)	0.004 (4)	0.012 (4)
C17	0.039 (4)	0.044 (4)	0.059 (5)	−0.009 (4)	0.011 (4)	0.003 (4)
C18	0.047 (5)	0.050 (5)	0.059 (5)	0.009 (4)	0.012 (4)	0.006 (4)
C19	0.055 (6)	0.095 (7)	0.070 (6)	−0.028 (5)	0.002 (5)	0.004 (5)
O3	0.128 (5)	0.052 (4)	0.081 (4)	−0.005 (4)	−0.005 (4)	−0.007 (3)
O4	0.075 (4)	0.083 (4)	0.059 (4)	−0.003 (3)	−0.006 (3)	−0.007 (3)
N2	0.056 (5)	0.066 (5)	0.094 (6)	0.018 (5)	0.003 (4)	−0.015 (5)
C20	0.095 (8)	0.069 (6)	0.072 (7)	0.006 (6)	0.025 (6)	−0.019 (6)
C21	0.106 (9)	0.094 (8)	0.069 (7)	0.037 (7)	0.021 (7)	0.009 (6)
C22	0.060 (7)	0.114 (8)	0.066 (7)	0.020 (7)	−0.037 (5)	0.003 (7)
C23	0.052 (6)	0.097 (7)	0.062 (6)	0.003 (5)	−0.012 (5)	−0.032 (6)
C24	0.040 (5)	0.053 (5)	0.031 (4)	0.010 (4)	0.015 (4)	0.007 (4)
C25	0.070 (6)	0.048 (5)	0.058 (5)	0.007 (4)	0.018 (4)	−0.007 (4)
C26	0.075 (6)	0.062 (5)	0.044 (4)	−0.010 (5)	−0.005 (4)	−0.001 (4)
C27	0.038 (5)	0.058 (5)	0.060 (5)	0.008 (4)	0.006 (4)	0.007 (4)
C28	0.022 (4)	0.057 (5)	0.061 (5)	0.007 (4)	−0.002 (4)	0.020 (4)
C29	0.042 (4)	0.034 (4)	0.069 (5)	−0.001 (3)	−0.010 (4)	0.004 (4)
C30	0.052 (5)	0.024 (4)	0.059 (5)	0.004 (3)	0.001 (4)	0.017 (3)
C31	0.074 (6)	0.059 (6)	0.080 (6)	−0.005 (5)	0.009 (5)	0.002 (5)
C32	0.049 (5)	0.036 (4)	0.098 (6)	−0.007 (4)	0.034 (5)	0.004 (4)
C33	0.050 (5)	0.055 (5)	0.040 (4)	0.006 (4)	−0.007 (4)	−0.004 (4)
C34	0.048 (5)	0.041 (4)	0.059 (5)	−0.002 (4)	−0.008 (4)	0.005 (4)
C35	0.037 (4)	0.051 (5)	0.055 (4)	0.003 (4)	0.005 (4)	0.002 (4)
C36	0.064 (5)	0.037 (4)	0.060 (5)	−0.003 (4)	−0.002 (4)	0.004 (4)
C37	0.044 (5)	0.056 (5)	0.066 (5)	0.009 (4)	0.014 (4)	0.017 (4)
C38	0.097 (8)	0.101 (7)	0.083 (7)	0.011 (7)	−0.016 (6)	−0.032 (6)

### Geometric parameters (Å, °)

**Table:**

O1—C6	1.217 (7)	O3—C25	1.219 (8)
O2—C14	1.355 (8)	O4—C33	1.383 (7)
O2—C19	1.436 (8)	O4—C38	1.480 (9)
N1—C1	1.320 (9)	N2—C24	1.325 (10)
N1—C5	1.323 (10)	N2—C20	1.351 (11)
C1—C2	1.357 (12)	C20—C21	1.393 (13)
C1—H1B	0.9300	C20—H20A	0.9300
C2—C3	1.404 (12)	C21—C22	1.355 (12)
C2—H2A	0.9300	C21—H21A	0.9300
C3—C4	1.359 (12)	C22—C23	1.368 (12)
C3—H3A	0.9300	C22—H22A	0.9300
C4—C5	1.415 (11)	C23—C24	1.352 (10)
C4—H4A	0.9300	C23—H23A	0.9300
C5—C6	1.509 (10)	C24—C25	1.516 (10)
C6—C7	1.443 (8)	C25—C26	1.455 (9)
C7—C8	1.361 (8)	C26—C27	1.328 (8)
C7—H7A	0.9300	C26—H26A	0.9300
C8—C9	1.453 (8)	C27—C28	1.442 (9)
C8—H8A	0.9300	C27—H27A	0.9300
C9—C10	1.349 (8)	C28—C29	1.398 (9)
C9—C18	1.437 (8)	C28—C37	1.433 (9)
C10—C11	1.396 (8)	C29—C30	1.391 (8)
C10—H10A	0.9300	C29—H29A	0.9300
C11—C12	1.411 (9)	C30—C31	1.397 (9)
C11—C16	1.430 (7)	C30—C35	1.414 (8)
C12—C13	1.376 (7)	C31—C32	1.379 (10)
C12—H12A	0.9300	C31—H31A	0.9300
C13—C14	1.407 (8)	C32—C33	1.426 (8)
C13—H13A	0.9300	C32—H32A	0.9300
C14—C15	1.390 (9)	C33—C34	1.342 (9)
C15—C16	1.403 (8)	C34—C35	1.427 (8)
C15—H15A	0.9300	C34—H34A	0.9300
C16—C17	1.405 (8)	C35—C36	1.410 (9)
C17—C18	1.404 (9)	C36—C37	1.340 (9)
C17—H17A	0.9300	C36—H36A	0.9300
C18—H18A	0.9300	C37—H37A	0.9300
C19—H19A	0.9600	C38—H38A	0.9600
C19—H19B	0.9600	C38—H38B	0.9600
C19—H19C	0.9600	C38—H38C	0.9600
			
C14—O2—C19	117.7 (7)	C33—O4—C38	116.0 (7)
C1—N1—C5	117.6 (8)	C24—N2—C20	114.5 (8)
N1—C1—C2	126.0 (9)	N2—C20—C21	122.9 (9)
N1—C1—H1B	117.0	N2—C20—H20A	118.5
C2—C1—H1B	117.0	C21—C20—H20A	118.5
C1—C2—C3	117.7 (9)	C22—C21—C20	119.3 (10)
C1—C2—H2A	121.2	C22—C21—H21A	120.4
C3—C2—H2A	121.2	C20—C21—H21A	120.4
C4—C3—C2	116.6 (9)	C21—C22—C23	117.9 (9)
C4—C3—H3A	121.7	C21—C22—H22A	121.1
C2—C3—H3A	121.7	C23—C22—H22A	121.1
C3—C4—C5	121.7 (9)	C24—C23—C22	119.1 (9)
C3—C4—H4A	119.2	C24—C23—H23A	120.4
C5—C4—H4A	119.2	C22—C23—H23A	120.4
N1—C5—C4	120.1 (8)	N2—C24—C23	125.8 (8)
N1—C5—C6	120.0 (8)	N2—C24—C25	114.8 (7)
C4—C5—C6	119.6 (8)	C23—C24—C25	119.2 (8)
O1—C6—C7	124.6 (7)	O3—C25—C26	121.5 (7)
O1—C6—C5	119.3 (7)	O3—C25—C24	118.0 (8)
C7—C6—C5	115.9 (7)	C26—C25—C24	120.5 (7)
C8—C7—C6	120.7 (7)	C27—C26—C25	121.9 (7)
C8—C7—H7A	119.7	C27—C26—H26A	119.1
C6—C7—H7A	119.7	C25—C26—H26A	119.1
C7—C8—C9	127.0 (7)	C26—C27—C28	125.8 (7)
C7—C8—H8A	116.5	C26—C27—H27A	117.1
C9—C8—H8A	116.5	C28—C27—H27A	117.1
C10—C9—C18	120.4 (7)	C29—C28—C37	116.1 (7)
C10—C9—C8	119.7 (7)	C29—C28—C27	119.9 (7)
C18—C9—C8	119.9 (6)	C37—C28—C27	123.8 (6)
C9—C10—C11	120.7 (7)	C30—C29—C28	122.7 (7)
C9—C10—H10A	119.6	C30—C29—H29A	118.7
C11—C10—H10A	119.6	C28—C29—H29A	118.7
C10—C11—C12	122.6 (6)	C29—C30—C31	120.4 (7)
C10—C11—C16	120.6 (6)	C29—C30—C35	119.8 (6)
C12—C11—C16	116.8 (7)	C31—C30—C35	119.8 (7)
C13—C12—C11	122.1 (7)	C32—C31—C30	120.0 (8)
C13—C12—H12A	119.0	C32—C31—H31A	120.0
C11—C12—H12A	119.0	C30—C31—H31A	120.0
C12—C13—C14	120.2 (7)	C31—C32—C33	119.2 (7)
C12—C13—H13A	119.9	C31—C32—H32A	120.4
C14—C13—H13A	119.9	C33—C32—H32A	120.4
O2—C14—C15	126.2 (7)	C34—C33—O4	124.2 (7)
O2—C14—C13	113.8 (7)	C34—C33—C32	122.6 (7)
C15—C14—C13	120.1 (7)	O4—C33—C32	113.2 (7)
C14—C15—C16	119.6 (7)	C33—C34—C35	118.4 (7)
C14—C15—H15A	120.2	C33—C34—H34A	120.8
C16—C15—H15A	120.2	C35—C34—H34A	120.8
C15—C16—C17	120.0 (7)	C36—C35—C30	117.3 (7)
C15—C16—C11	121.3 (6)	C36—C35—C34	122.8 (6)
C17—C16—C11	118.7 (7)	C30—C35—C34	120.0 (7)
C18—C17—C16	119.8 (7)	C37—C36—C35	122.6 (8)
C18—C17—H17A	120.1	C37—C36—H36A	118.7
C16—C17—H17A	120.1	C35—C36—H36A	118.7
C17—C18—C9	119.7 (6)	C36—C37—C28	121.5 (7)
C17—C18—H18A	120.2	C36—C37—H37A	119.2
C9—C18—H18A	120.2	C28—C37—H37A	119.2
O2—C19—H19A	109.5	O4—C38—H38A	109.5
O2—C19—H19B	109.5	O4—C38—H38B	109.5
H19A—C19—H19B	109.5	H38A—C38—H38B	109.5
O2—C19—H19C	109.5	O4—C38—H38C	109.5
H19A—C19—H19C	109.5	H38A—C38—H38C	109.5
H19B—C19—H19C	109.5	H38B—C38—H38C	109.5
			
C5—N1—C1—C2	−1.6 (16)	C24—N2—C20—C21	−6.1 (15)
N1—C1—C2—C3	5.3 (16)	N2—C20—C21—C22	9.5 (18)
C1—C2—C3—C4	−5.7 (14)	C20—C21—C22—C23	−5.7 (18)
C2—C3—C4—C5	3.1 (15)	C21—C22—C23—C24	−0.5 (17)
C1—N1—C5—C4	−1.4 (14)	C20—N2—C24—C23	−0.7 (12)
C1—N1—C5—C6	−175.6 (8)	C20—N2—C24—C25	−175.0 (8)
C3—C4—C5—N1	0.5 (15)	C22—C23—C24—N2	4.0 (14)
C3—C4—C5—C6	174.7 (9)	C22—C23—C24—C25	178.1 (8)
N1—C5—C6—O1	−175.6 (8)	N2—C24—C25—O3	175.7 (7)
C4—C5—C6—O1	10.2 (12)	C23—C24—C25—O3	1.0 (11)
N1—C5—C6—C7	−1.1 (11)	N2—C24—C25—C26	−5.9 (10)
C4—C5—C6—C7	−175.4 (7)	C23—C24—C25—C26	179.4 (7)
O1—C6—C7—C8	13.6 (11)	O3—C25—C26—C27	−20.5 (12)
C5—C6—C7—C8	−160.5 (7)	C24—C25—C26—C27	161.1 (7)
C6—C7—C8—C9	176.9 (7)	C25—C26—C27—C28	−178.6 (6)
C7—C8—C9—C10	−155.4 (7)	C26—C27—C28—C29	159.6 (7)
C7—C8—C9—C18	24.3 (11)	C26—C27—C28—C37	−16.2 (12)
C18—C9—C10—C11	−3.6 (11)	C37—C28—C29—C30	0.9 (10)
C8—C9—C10—C11	176.1 (6)	C27—C28—C29—C30	−175.2 (6)
C9—C10—C11—C12	−179.3 (7)	C28—C29—C30—C31	179.0 (7)
C9—C10—C11—C16	3.7 (10)	C28—C29—C30—C35	0.0 (10)
C10—C11—C12—C13	−177.4 (6)	C29—C30—C31—C32	−179.6 (7)
C16—C11—C12—C13	−0.2 (10)	C35—C30—C31—C32	−0.5 (11)
C11—C12—C13—C14	−0.6 (11)	C30—C31—C32—C33	−0.3 (12)
C19—O2—C14—C15	5.2 (10)	C38—O4—C33—C34	−4.3 (10)
C19—O2—C14—C13	−174.2 (6)	C38—O4—C33—C32	173.8 (6)
C12—C13—C14—O2	−179.8 (6)	C31—C32—C33—C34	0.0 (11)
C12—C13—C14—C15	0.8 (11)	C31—C32—C33—O4	−178.1 (7)
O2—C14—C15—C16	−179.4 (6)	O4—C33—C34—C35	179.1 (6)
C13—C14—C15—C16	−0.1 (10)	C32—C33—C34—C35	1.2 (10)
C14—C15—C16—C17	179.5 (7)	C29—C30—C35—C36	−1.1 (9)
C14—C15—C16—C11	−0.8 (10)	C31—C30—C35—C36	179.9 (7)
C10—C11—C16—C15	178.1 (6)	C29—C30—C35—C34	−179.2 (6)
C12—C11—C16—C15	0.9 (9)	C31—C30—C35—C34	1.7 (10)
C10—C11—C16—C17	−2.2 (9)	C33—C34—C35—C36	179.9 (7)
C12—C11—C16—C17	−179.4 (6)	C33—C34—C35—C30	−2.1 (10)
C15—C16—C17—C18	−179.7 (6)	C30—C35—C36—C37	1.3 (11)
C11—C16—C17—C18	0.6 (10)	C34—C35—C36—C37	179.4 (7)
C16—C17—C18—C9	−0.5 (10)	C35—C36—C37—C28	−0.4 (11)
C10—C9—C18—C17	2.0 (11)	C29—C28—C37—C36	−0.7 (10)
C8—C9—C18—C17	−177.7 (6)	C27—C28—C37—C36	175.3 (7)

### Hydrogen-bond geometry (Å, °)

**Table:**

*D*—H···*A*	*D*—H	H···*A*	*D*···*A*	*D*—H···*A*
C38—H38B···O1^i^	0.96	2.53	3.356 (10)	143

Symmetry codes: (i) *x*−1/2, −*y*+1, *z*.
